# Comparison of neoadjuvant chemoimmunotherapy and chemotherapy alone for resectable stage III non-small cell lung cancer: a real-world cohort study

**DOI:** 10.3389/fimmu.2023.1343504

**Published:** 2023-12-22

**Authors:** Sihao Zhou, Yi Liu, Kejun Liu, Junkai Zhang, Hanlin Liang, Yingmeng Wu, Hongyu Ye, Yi Liang, Jingjing Zhang, Weizhao Huang

**Affiliations:** ^1^ Department of Cardiothoracic Surgery, Zhongshan City People’s Hospital, Zhongshan, China; ^2^ Department of Pulmonary Oncology, Zhongshan City People’s Hospital, Zhongshan, China; ^3^ Department of Radiotherapy, Zhongshan City People’s Hospital, Zhongshan, China

**Keywords:** non-small-cell lung cancer, neoadjuvant therapy, chemotherapy, immunotherapy, efficacy, safety

## Abstract

**Background:**

We compared the real-world efficacy and safety of neoadjuvant chemoimmunotherapy to chemotherapy alone in patients with stage III non-small-cell lung cancer (NSCLC).

**Participants and methods:**

A total of 59 consecutive patients were finally selected and divided into two groups: the neoadjuvant chemotherapy group (n = 33) and the neoadjuvant chemoimmunotherapy group (n = 26). The primary endpoint was disease-free survival (DFS). The secondary endpoints were pathological response, clinical response, and adverse events. All patients were followed up to collect perioperative pathology and clinical data.

**Results:**

The objective response rate (ORR), pathological complete response (pCR), and major pathological response (MPR) were significantly higher in the neoadjuvant chemoimmunotherapy group than in the neoadjuvant chemotherapy group (73.1% vs. 45.5%, 34.6% vs. 3.0%, and 65.3% vs. 15.1%, respectively; *P* < 0.05*)*. There was no statistically significant difference in disease-free survival between the neoadjuvant chemoimmunotherapy and neoadjuvant chemotherapy groups (*P* = 0.129). Patients in the neoadjuvant chemoimmunotherapy group had a higher rate of tumor regression than those in neoadjuvant chemotherapy group (37.0% [25 patients] vs. 29.0% [33 patients], *P =* 0.018). However, no discernible correlation between MPR achievement and the degree of tumor shrinkage was observed in either group (*P* > 0.05). The cumulative MPR rates were 42.3, 50, and 65.3% for 2, 3, and ≥ 4 cycles, respectively, in the neoadjuvant chemoimmunotherapy group and 9.1, 12.1, and 15.1% for ≤ 2, 3, and ≥ 4 cycles, respectively, in the neoadjuvant chemotherapy group. Moreover, No statistical difference was observed between the two groups regarding postoperative complications, resection range, operation time, surgical method, and extent of resection (*P* > 0.05). Although the incidence of grades III–IV adverse events was higher in the neoadjuvant chemotherapy group than in the neoadjuvant chemoimmunotherapy group (33.3% vs. 4.6%, *P* = 0.042), there was no significant difference in the incidence of adverse events between the two groups (64.6% vs. 83.6%, *P* = 0.072).

**Conclusion:**

In stage III NSCLC, neoadjuvant chemoimmunotherapy achieved higher pathological and clinical remission rates than chemotherapy alone, with compromising safety, making it an attractive choice for neoadjuvant therapy.

## Introduction

Currently, the treatment of stage III non-small-cell lung cancer (NSCLC) remains a significant challenge. Existing literature suggests that the 5-year survival rate following surgery in patients with stage III NSCLC is only 13–36%, whereas that for surgery combined with adjuvant radiotherapy and for chemotherapy alone is merely 20 and 45%, respectively (1, 2). The National Comprehensive Cancer Network guidelines of the United States have suggested the use of nivolumab monoclonal antibody along with platinum-based doublet chemotherapy as a neoadjuvant therapy regimen for NSCLC (3). However, studies specifically focusing on the use of neoadjuvant chemoimmunotherapy for stage III NSCLC are lacking. To the best of our knowledge, there are also currently no studies discussing the derived patterns of major pathological responses (MPRs) following neoadjuvant chemoimmunotherapy. In this real-world study, we examined the clinical and pathological data of patients with stage III NSCLC who underwent pre-operative neoadjuvant therapy. We aimed to compare the safety and efficacy of neoadjuvant chemoimmunotherapy with those of neoadjuvant chemotherapy, paying special attention to the pattern of MPR occurrence, which could serve as a beneficial reference for selecting the most appropriate neoadjuvant therapy for stage III NSCLC.

## Patients and methods

This real-world study included patients diagnosed with stage III NSCLC between October 2017 and October 2023, who were divided into two groups: the neoadjuvant chemotherapy group and the neoadjuvant chemoimmunotherapy group. This study was conducted in accordance with the Declaration of Helsinki. All patients provided informed consent.

The inclusion criteria were as follows: 1) stage III diagnosis of NSCLC via imaging and cytological examination, with all tumors identified as primary lung cancer; 2) preoperative assessment by multiple senior surgeons, deeming the lesion resectable, despite a large tumor volume involving the carina, bronchus, or pulmonary vessels, with hilar or mediastinal lymph node metastases; 3) Karnofsky performance status score ≥ 80, indicating the capacity to tolerate neoadjuvant therapy; 4) normal liver and kidney functions; 5) anticipated survival time of > 3 months; 6) sufficient pulmonary function for the expected pneumonectomy; and 7) negative for *EGFR* and *ALK* gene mutations.

The exclusion criteria were as follows: 1) distant metastasis or surgical contraindications; 2) dysfunction of the liver, kidney, or other organs; 3) autoimmune diseases (e.g., diseases due to human immunodeficiency virus) or long-term use of immunosuppressive drugs; and 4) intolerance to immunotherapy or chemotherapy.

Patients treated with a combination of programmed cell death-1 monoclonal antibody and platinum-based doublet chemotherapy were included in the immunochemotherapy group, whereas those treated only with platinum-based doublet chemotherapy were included in the chemotherapy group. The immunotherapy drugs included pembrolizumab, tislelizumab, sintilimab, camrelizumab, and nivolumab. Chemotherapy was based on the following first-line regimen for advanced NSCLC.

Four patients underwent treatment with gemcitabine 1000 mg/m^2^ on days 1 and 8 and platinum-based therapy, whereas two received etoposide 100 mg/m^2^ on days 1–3 and platinum-based therapy. A 21-day cycle was followed for both immunotherapy and chemotherapy, in which the tumor efficacy was assessed every two cycles. Regular examinations via hematology and imaging were performed during the treatment period.

Chest tomography or positron emission tomography-computed tomography reassessment was performed every two cycles, and the efficacy of the treatment was assessed according to the Response Evaluation Criteria in Solid Tumors, version 1.1 (4). Pathological complete response (pCR) was defined as neoadjuvant therapy-induced tumor regression with no visible residual tumor on pathological examination. A major pathological response (MPR) was defined as neoadjuvant therapy-induced tumor regression with ≤ 10% residual tumor on pathological examination (MPR includes pCR) (5, 6). Non-MPR was defined as a major pathological response not being reached.

The time to surgery after neoadjuvant therapy was taken as the duration from treatment completion to surgical intervention. Any adverse events (AEs) occurring from the initiation of the medication under study to 1 month after treatment completion, regardless of any causal relationship with the trial drugs, were considered AEs. AEs were evaluated using the Common Toxicity Criteria Document for Adverse Events, version 5.0 of the US National Cancer Institute.

Patients were monitored every 3 months until October 2023. The primary endpoint was disease-free survival (DFS), which was defined as the period from surgery to the discovery of disease recurrence or death. The secondary endpoints were pathological response, clinical response, and AEs.

### Statistical analysis

All statistical analyses were performed using Statistical Product and Service Solutions, version 25.0. Count data are presented as numbers and percentages. Intergroup comparisons were conducted using the χ^2^ test. Independent sample t-tests were used for measurement information. The effect size was estimated according to the relative risk and corresponding 95% confidence intervals, survival analysis comparisons using the Kaplan–Meier survival curve analysis, and correlation analysis using point-biserial correlation. The significance levels for all tests were established at α = 0.05.

## Results

### Baseline characteristics of the patients

**Figure 1:**
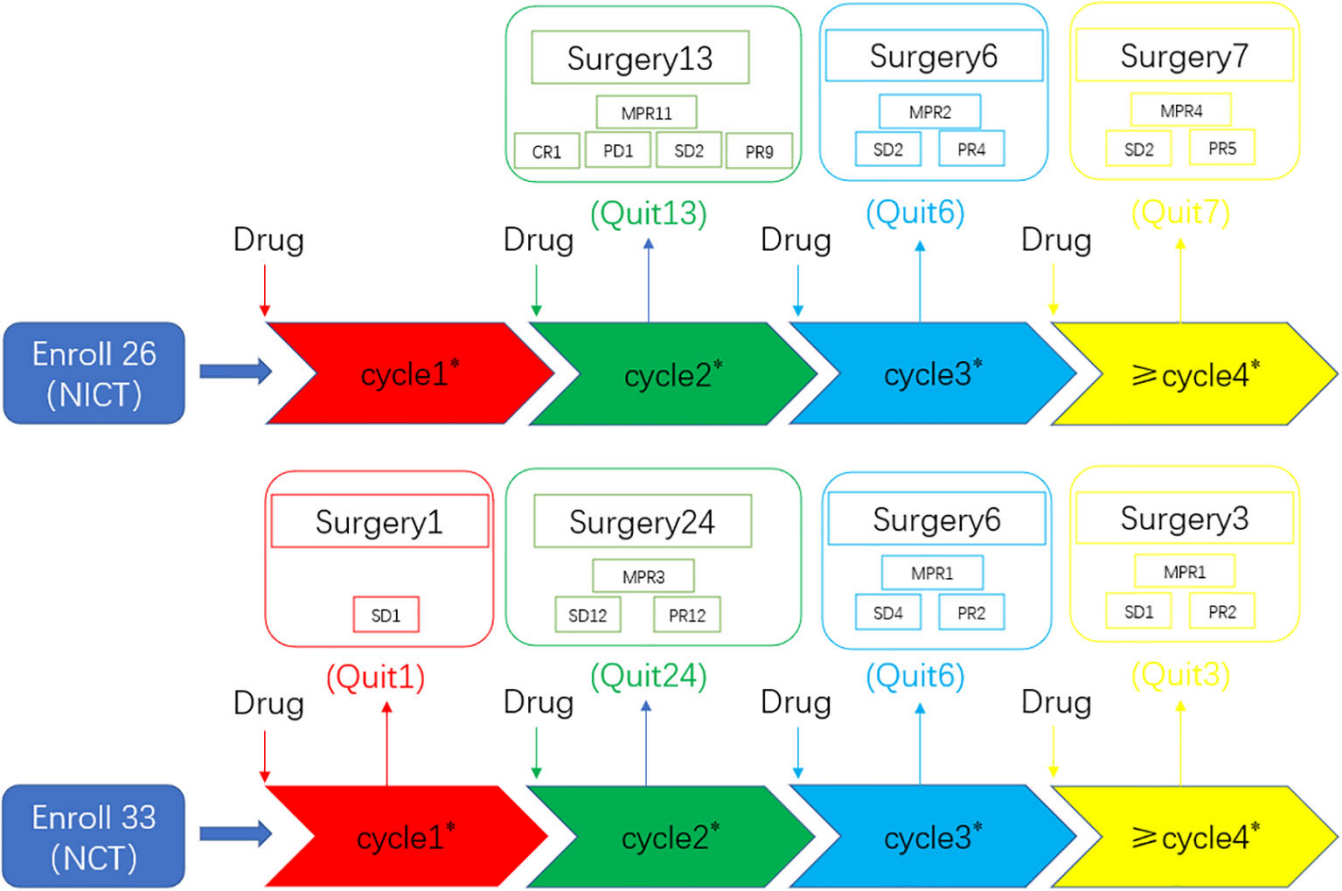
Overview of the treatment processes. A total of 59 consecutive patients were ultimately selected and divided into two groups: the neoadjuvant chemotherapy group (n = 33) and the neoadjuvant chemoimmunotherapy group (n = 26). All patients underwent randomization and received a neoadjuvant treatment followed by surgery. CR, complete response; NCT, neoadjuvant chemotherapy group; NICT, neoadjuvant chemoimmunotherapy group; MPR, major pathological response; PD, progressive disease; PR, partial response; SD, stable disease. Numeric values represent the number of patients. Number * represents the number of neoadjuvant therapy cycles. “Quit” signifies patients who underwent surgery after completing neoadjuvant therapy.

The neoadjuvant therapy groups comprised 59 patients who fulfilled the defined inclusion criteria. **Figure 1** shows the treatment processes followed in the two groups, and **Table 1** shows the clinical data of the enrolled patients. General clinical data comparison revealed no statistically significant difference between the two groups (*P* > 0.05).

**Table 1:** Baseline characteristics of the patients (n = 59).

Characteristics	Neoadjuvant chemoimmunotherapy group (n = 26)	Neoadjuvant chemotherapy group (n = 33)	χ^2^ value	*P*-value
Sex			0.617	0.432
Male	24 (92.4%)	27 (81.9%)		
Female	2 (7.6%)	6 (18.1%)		
Age			0.998	0.318
≤60 years	16 (61.6%)	16 (48.4%)		
>60 years	10 (38.4%)	17 (51.6%)		
Body mass index (BMI)			0.032	0.859
<24 kg/m^2^	14 (53.9%)	17 (51.6%)		
≥24 kg/m^2^	12 (46.1%)	16 (48.4%)		
TTS			1.562	0.211
≤30	12 (46.1%)	10 (30.3%)		
>30	14 (53.9%)	23 (69.7%)		
Clinical TNM stage			0.001	0.976
IIIA	19 (73.1%)	24 (72.8%)		
IIIB	7 (26.9%)	9 (27.2%)		
Treatment cycles			4.097	0.129
≤2	13 (50%)	24 (72.8%)		
3	6 (23.1%)	6 (18.1%)		
≥4	7 (26%)	3 (9.1%)		
Histologic type			0.280	0.869
Squamous cell carcinoma	12 (46.1%)	13 (39.5%)		
Adenocarcinoma	10 (38.4%)	14 (42.4%)		
Others	4 (15.5%)	6 (18.1%)		
CN			1.273	0.529
N0	2 (7.6%)	3 (9.1%)		
N1	8 (30.8%)	6 (18.1%)		
N2	16 (61.6%)	24 (72.8%)		
PD-L1 expression			2.200	0.333
<1%	16 (61.5%)	14 (42.4%)		
≥1–50%	8 (30.8%)	16 (48.5%)		
≥50%	2 (7.7%)	3 (9.1%)		

CN, lymph node staging before neoadjuvant therapy; PD-L1, programmed cell death ligand 1; TTS, time to surgery - indicating the time from the end of neoadjuvant therapy to surgery.

### Comparison of the efficacy of neoadjuvant therapies

**Figure 2:**
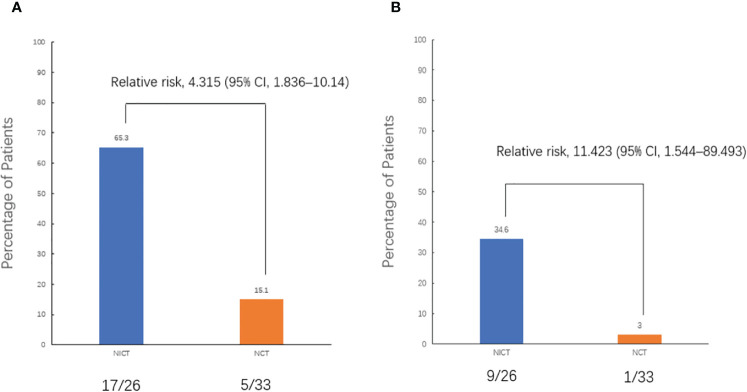
Pathological response. **(A)** Comparison of the major pathological response (MPR) rates between the two groups. **(B)** Comparison of the pathological complete response (pCR) rates between the two groups. All patients underwent randomization and received a neoadjuvant treatment. The assessment of pathological response was valid for all patients who underwent surgery (59 patients). pCR was defined as 0% residual tumor on pathological examination, and major pathological response (MPR) was defined as neoadjuvant therapy-induced tumor regression with ≤ 10% residual tumor on pathological examination. CI, confidence interval; NCT, neoadjuvant chemotherapy group; NICT, neoadjuvant chemoimmunotherapy group.

**Figure 3:**
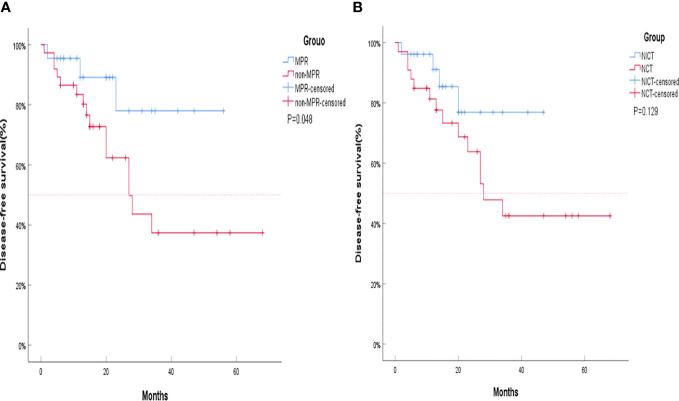
Kaplan–Meier curves for survival stratified by disease-free survival (DFS). **(A)** DFS stratified by treatment plan. **(B)** DFS stratified by major pathological response (MPR). The MPR group and non-MPR group are represented by Kaplan–Meier curves for survival stratified by DFS. The neoadjuvant chemotherapy group (NCT) and neoadjuvant chemoimmunotherapy (NICT) group are represented by Kaplan–Meier curves for survival stratified by DFS. Although the DFS of the NICT group and the NCT group did not show statistical differences, the separation of the two curves was already clear, and the DFS of the NICT group showed a trend of benefit. The shorter curve of the NICT group could be attributed to its later development compared to NCT, resulting in insufficient enrollment and follow-up time, which is also the reason that significant differences were not achieved. NCT, neoadjuvant chemotherapy group; NICT, neoadjuvant chemoimmunotherapy group; MPR, major pathological response.

The objective response rate (ORR) of the neoadjuvant chemoimmunotherapy group was significantly higher than that of the neoadjuvant chemotherapy group (*P* = 0.033). Similarly, the pCR and MPR rates were significantly higher in the neoadjuvant chemoimmunotherapy group than in the neoadjuvant chemotherapy group (*P* = 0.004, *P* = 0.004). However, no statistical difference was observed in terms of complete remission (CR), partial remission (PR), stable disease (SD), progressive disease (PD), and disease control rate (DCR) between the two groups (*P* > 0.05). The postoperative occurrence of positive lymph node metastasis, pleural invasion, vascular invasion, and nerve invasion revealed no statistical difference between the groups (*P* > 0.05). These details are presented in **Table 2**. A major pathological response occurred in 19 of the 26 patients in the neoadjuvant chemoimmunotherapy group and in 5 of the 33 patients in the neoadjuvant chemotherapy group (relative risk [RR]: 4.315; 95% confidence interval [CI]: 1.836 to 10.142) (**Figure 2A**). A pCR occurred in 9 of 26 patients in the neoadjuvant chemoimmunotherapy group and in 1 of 33 patients in the neoadjuvant chemotherapy group (RR: 11.423; 95% CI: 1.544 to 84.493) (**Figure 2B**). The MPR group included three cases of postoperative recurrence, whereas the non-MPR group included 15 cases of postoperative recurrence. A statistically significant difference was observed in DFS postoperatively between the MPR and non-MPR groups (*P* = 0.048; **Figure 3A**). The neoadjuvant chemoimmunotherapy group included four cases of postoperative recurrence, whereas the neoadjuvant chemotherapy group recorded 14 cases. No statistical difference was observed in DFS between these groups postoperatively (*P* = 0.129; **Figure 3B**).

**Table 2:** Comparison of the efficacy after neoadjuvant therapy in 59 patients with non-small-cell lung cancer.

Efficacy evaluation	Neoadjuvant chemoimmunotherapy group (n = 26)	Neoadjuvant chemotherapy group (n = 33)	χ^2^ value	*P*-value
CR	1 (3.8%)	0	0.015	0.904
PR	18 (69.3%)	15 (45.5%)	3.335	0.068
SD	6 (23.1%)	18 (54.5)	5.968	0.015
PD	1 (3.8%)	0	0.015	0.904
ORR	19 (73.1%)	15 (45.5%)	4.544	0.033
DCR	25 (96.2%)	33 (100%)	0.015	0.904
pCR	9 (34.6%)	1 (3.0%)	8.185	0.004
MPR	17 (65.3%)	5 (15.1%)	15.693	0.000
ypN			0.135	0.713
N (+)	6 (23.1%)	9 (27.3%)		
N (-)	20 (76.9%)	24 (72.7%)		
ypTNM			10.328	0.016
Stage 0	9 (34.6%)	1 (3.0%)		
Stage I	6 (23.1%)	12 (36.4%)		
Stage II	5 (19.2%)	9 (27.3%)		
Stage III	6 (23.1%)	11 (33.3%)		
Pleural invasion	1 (3.8%)	3 (9.0%)	0.075	0.784
Vascular invasion	1 (3.8%)	6 (18.0%)	1.652	0.199
Nerve invasion	1 (3.8%)	1 (3.0%)	0.000	1.000

CR, complete response; DCR, disease control rate; MPR, major pathological response; ORR, objective response rate; pCR, pathological complete response; PD, progressive disease; PR, partial response; SD, stable disease; ypN, post-neoadjuvant pathological lymph node staging; ypTNM, post-neoadjuvant pathologic stages.

**Figure 4:**
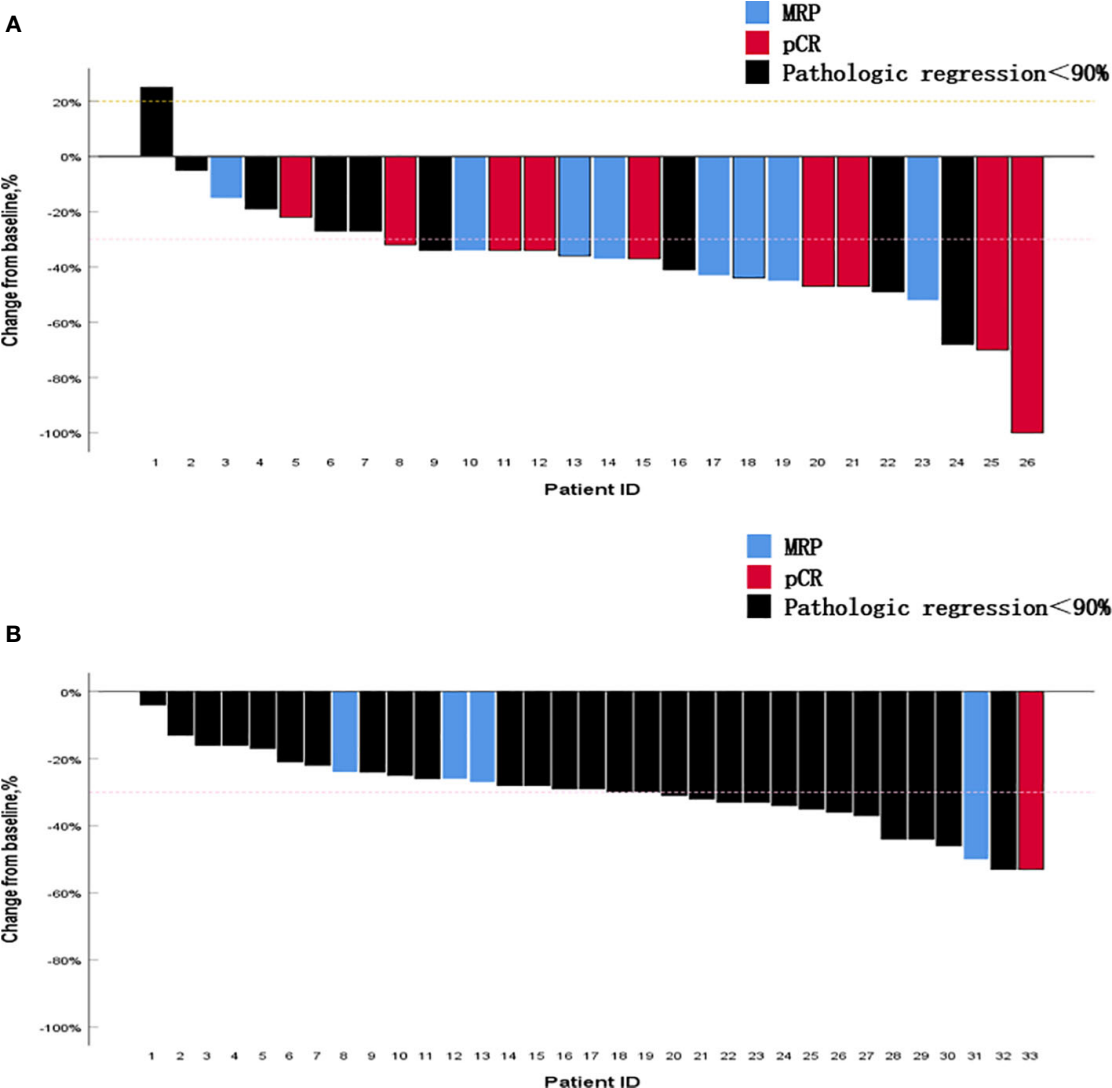
Percentage change in the diameter of the maximum target lesion from baseline. **(A)** Neoadjuvant chemoimmunotherapy group and **(B)** neoadjuvant chemotherapy group. The figure shows the change in the maximum tumor diameter after neoadjuvant therapy in all patients. It is noteworthy that most tumors that obtained pathological complete response (pCR) or major pathological response (MPR) did not exhibit corresponding complete regression in their imaging findings. MPR, major pathological response; pCR, pathological complete response.

**Figure 5:**
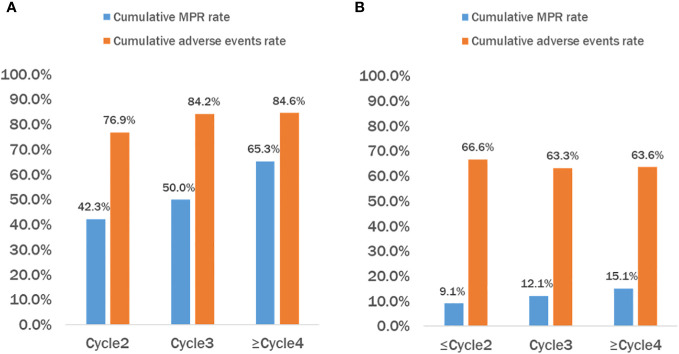
Cumulative MPR rate and cumulative adverse events rate of patients in the chemoimmunotherapy and chemotherapy groups. **(A)** Neoadjuvant chemoimmunotherapy (NICT) group and **(B)** neoadjuvant chemotherapy (NCT) group. It can be observed that as the treatment cycle increases, the cumulative major pathological response (MPR) rate in the NICT group also continues to rise, but the incidence of cumulative adverse events reaches a plateau after 3–4 courses of treatment. While the cumulative MPR rate in the NCT group also increased, it was significantly lower than that in the NICT group, and the cumulative adverse events continued to increase accordingly. NCT, neoadjuvant chemotherapy group; NICT, neoadjuvant chemoimmunotherapy group; MPR, major pathological response.

All patients in the neoadjuvant chemotherapy group experienced tumor regression (tumor regression rate: 4–53%; **Figure 4A**). The neoadjuvant chemoimmunotherapy group included 25 cases of tumor regression (tumor regression rate: 5–100%; **Figure 4B**). The tumor regression rate was higher in the neoadjuvant chemoimmunity group than in the neoadjuvant chemotherapy group (37.0% vs. 29.0%, *P* = 0.018; **Table 3**). The achievement of MPR in both groups showed no correlation with the degree of tumor shrinkage (*P* > 0.05; **Table 4**). The cumulative MPR rates were 42.3%, 50%, and 65.3%, while the cumulative adverse event rates were 76.9%, 84.2%, and 84.6%, for 2, 3, and ≥ 4 cycles, respectively, in the neoadjuvant chemoimmunotherapy group (**Figure 5A**). In the neoadjuvant chemotherapy group, these rates were 9.1%, 12.1%, and 15.1% (MPR rate), and 66.6%, 63.6%, and 63.6% (cumulative adverse event rate) for ≤ 2, 3, and ≥ 4 cycles, respectively (**Figure 5B**). The cumulative MPR rate of patients in the neoadjuvant chemoimmunotherapy group continuously increased with the number of treatment courses.

**Table 3:** Comparison of the tumor regression rates between the two groups.

	Neoadjuvant chemoimmunotherapy group	Neoadjuvant chemotherapy group	*P*-value
Tumor regression rate	5–100%	4–53%	
Mean tumor regression rate	37.0%	29.0%	0.018

**Table 4:** Correlation analysis between the degree of tumor shrinkage and the major pathological response.

Group	Point-biserial correlation	*P*-value
Neoadjuvant chemoimmunotherapy group	−0.344	0.095
Neoadjuvant chemotherapy group	−0.221	0.216

### Comparison of the perioperative indicators of neoadjuvant therapies

All 59 patients underwent surgical treatment. Analysis of postoperative complications, resection range, operation time, surgical method, and extent of resection exhibited no statistical difference between the two groups (*P* > 0.05; **Table 5**).

**Table 5:** Comparison of the perioperative indicators in 59 patients with non-small-cell lung cancer.

Perioperative indicators	Neoadjuvant chemoimmunotherapy group (n = 26)	Neoadjuvant chemotherapy group (n = 33)	χ^2^ value	*P*-value
Postoperative complications			2.136	0.144
Yes	7 (26.7%)	15 (45.4%)		
No	19 (73.3%)	18 (54.6%)		
Resection range			0.520	0.471
Lobectomy	21 (80.7%)	24 (72.7%)		
Extended resection	5 (19.3%)	9 (27.3%)		
Operation time			0.494	0.482
≤200 min	11 (42.3%)	17 (51.5%)		
>200 min	15 (57.7%)	16 (48.5%)		
Surgical method			0.000	1.000
Open surgery	0	1 (3.0%)		
VATS	26 (100%)	32 (97.0%)		
Extent of resection				
Non-R0	0	1 (3.0%)	0.000	1.000
R0	26 (100%)	32 (97.0%)		

Extended resection includes combined lobectomy, bronchoplasty, sleeve lobectomy, vena cava replacement, and pulmonary arterioplasty.

VATS, video-assisted thoracoscopic surgery.

### Comparison of the AEs of neoadjuvant therapies

**Table 6** presents the AEs of all patients in the study. The occurrence of grades III–V AEs was significantly higher in the neoadjuvant chemotherapy group than in the neoadjuvant chemoimmunotherapy group (*P* = 0.042). Blood system-related AEs were the most common in both groups (neoadjuvant chemoimmunotherapy group: 95.4%; neoadjuvant chemotherapy group: 57.1%). However, the incidence of AEs, such as low leukocyte count, liver function abnormalities, and nausea and vomiting, did not differ significantly between the two groups (*P* = 0.072; **Table 7**).

**Table 6:** Adverse events in 59 patients with non-small-cell lung cancer.

Adverse events	Neoadjuvant chemoimmunotherapy group (n = 26)		Neoadjuvant chemotherapy group (n = 33)	
Grading	I–II	III–IV	I–II	III–IV
Blood system abnormalities	19	1	7	5
Anemia	15	0	7	0
Low leukocyte count	6	0	3	0
Low platelet count	0	0	0	2
Neutropenia	1	1	1	3
Digestive system abnormalities	9	0	10	1
Abnormal liver functions	5	0	2	1
Nausea and vomiting	6	0	9	0
Abnormal thyroid functions	2	0	0	0
Abnormal kidney functions	1	0	0	0
Pneumonia	2	0	0	1
Fever	1	0	2	0
Hypoproteinemia	6	0	1	0
Itching	1	0	0	0

**Table 7:** Comparison of the adverse reactions in 59 patients with non-small-cell lung cancer.

Variables	Neoadjuvant chemoimmunotherapy group (n = 26)	Neoadjuvant chemotherapy group (n = 33)	χ^2^ value	*P*-value
Adverse events			3.238	0.072
Yes	22 (84.6%)	21 (63.6%)		
No	4 (15.4%)	12 (36.4%)		
Adverse events grading			4.133	0.042
I–II	21 (95.4%)	14 (66.7%)		
III–IV	1 (4.6%)	7 (33.3%)		
Blood system				
Yes	20 (90.8%)	12 (57.1%)	6.435	0.011
No	2 (9.2)	9 (42.9%)		
Anemia			5.222	0.022
Yes	15 (68.1%)	7 (33.3%)		
No	7 (31.9%)	14 (66.7%)		
Low leukocyte count			0.451	0.502
Yes	6 (27.2%)	3 (14.2%)		
No	16 (72.8%)	18 (85.8%)		
Digestive system abnormalities			0.568	0.451
Yes	9 (40.9%)	11 (52.3%)		
No	13 (59.1%)	10 (47.7%)		
Abnormal liver functions			0.102	0.750
Yes	5 (22.7%)	3 (14.2%)		
No	17 (77.3%)	18 (85.8%)		
Nausea and vomiting			1.149	0.284
Yes	6 (27.2%)	9 (42.9%)		
No	16 (72.8%)	12 (57.1%)		

## Discussion

Several studies (7, 8) have shown that chemotherapy not only induces immunogenic cell death (ICD) in tumor cells, leading to an anti-tumor immune response, but also improves the tumor microenvironment by removing some of the immunosuppressive cells and increasing the number of immune cells with anti-tumor effects, thus enhancing the anti-tumor immune response from the surface. Therefore, the combination of neoadjuvant immunotherapy with chemotherapy may be effective in treating cancer. The results of several clinical studies of neoadjuvant chemoimmunotherapy versus neoadjuvant chemotherapy in stage I–III NSCLC have shown that the pCR and MPR rates of the neoadjuvant chemoimmunotherapy group were significantly higher than those of the neoadjuvant chemotherapy alone group (9–11); however, most of these studies enrolled patients with a wider range of clinical stages, including stages I–III. The present study enrolled a better homogeneity of patients with stage III NSCLC, and used a variety of chemoimmunotherapy combination regimens, which is closer to the real-world situation, and also achieved better efficacy. Our findings are consistent with those of a prospective trial of neoadjuvant nivolumab in combination with platinum-based two-agent chemotherapy versus chemotherapy alone in stage III lung cancer (NCT03838159) (12). The 2014 (University of Texas Anderson Lung Cancer Study Group) Oncology Expert Consensus (13) concluded that the MPR is associated with long-term prognosis of patients with lung cancer. In our study, 59 patients were divided into MPR and non-MPR groups, and the DFS of patients in the MPR group was significantly higher than that in the non-MPR group, suggesting that an MPR indicates a good prognosis. Results from clinical studies have indicated that there is generally a higher MPR rate in neoadjuvant chemoimmunotherapy groups compared to neoadjuvant chemotherapy groups. Additionally, the DFS of neoadjuvant chemoimmunotherapy groups has been shown to be significantly better than that of neoadjuvant chemotherapy groups (9, 10, 14, 15). However, in the present study, we found that the DFS of the neoadjuvant chemoimmunotherapy group was not significantly higher than that of the neoadjuvant chemotherapy group (*P* = 0.129). This may be attributed to the short follow-up time in this study, as well as the fact that the neoadjuvant chemoimmunotherapy group had not yet reached the median follow-up time, which led to immature DFS data; however, the results indicated that the 2-year DFS rate of the neoadjuvant chemoimmunotherapy group was 76.9% higher than that of the neoadjuvant chemotherapy group, at 63.8%, suggesting that patients may potentially benefit from neoadjuvant chemoimmunotherapy. Therefore, it can be assumed that the new modality of neoadjuvant chemoimmunotherapy has better disease outcomes compared to neoadjuvant chemotherapy alone in stage III NSCLC, and may show better efficacy in terms of pathologic remission and thus a better prognosis.

The main purpose of neoadjuvant therapy is to shrink the tumor, reduce the stage, and improve the resectability of surgery. In this study, we found that the tumor regression rate of the neoadjuvant chemoimmunotherapy group was significantly higher than that of the neoadjuvant chemotherapy group, suggesting that neoadjuvant chemoimmunotherapy can more effectively achieve tumor shrinkage, which is conducive to radical surgical resection. The CheckMate-816 (11) results also suggested that the neoadjuvant chemoimmunotherapy group had a higher rate of R0 resection, a higher percentage of minimally invasive surgeries, and a lower proportion of total lung resections. By analyzing the correlation between patients’ tumor regression rate and MPR, it was found that there was no significant correlation between the two, which is similar to the findings of a phase 2 clinical trial (NCT04304248) (16). This suggests that although the tumor regression is not significant on imaging, the actual residual tumor activity may have been significantly reduced. Therefore, for patients undergoing neoadjuvant treatment with chemotherapy plus immunotherapy, aggressive surgery should be performed if the tumor is considered potentially resectable and more accurate evaluation methods should be adopted.

The duration of neoadjuvant immunotherapy is currently inconclusive, with most studies generally opting for 2–4 cycles. The current CheckMate-159 trial (17) gave two cycles of treatment with an MPR of 45%, and the NADIM study (18) gave three cycles of treatment with an MPR of 83%. The present study found that the cumulative MPR rate of patients in the neoadjuvant chemoimmunotherapy group increased with the number of treatment cycles. However, the cumulative adverse event rate of patients also increased in both groups. Due to the small sample size, our findings require further confirmation regarding whether: 1) extending the number of cycles improves the MPR rate and 2) the balance between the patient’s tolerance and the number of cycles of treatment needs exploration.

Several studies have found that surgical resection of the lungs after neoadjuvant immunotherapy is feasible and does not delay the timing to surgery. In addition, the overall perioperative outcome is relatively safe, similar to that of patients receiving neoadjuvant platinum-based chemotherapy (19, 20). Similarly, the findings of the present study suggested that neoadjuvant chemoimmunotherapy did not significantly increase surgery-related AEs and that surgery performed after neoadjuvant chemoimmunotherapy had a safety profile similar to neoadjuvant chemotherapy. Theoretically, the addition of immunotherapy inevitably increases drug-related AEs. In this study, the incidence of AEs was slightly higher in the neoadjuvant chemoimmunotherapy group, but grade I–II AEs were predominant, and the incidence of grade III and above AEs was much lower than that in the neoadjuvant chemotherapy group, which had less impact on the subsequent surgical treatment. According to a study, neoadjuvant chemoimmunotherapy not only has a synergistic anti-tumor effect but may also be caused by different antitumor mechanisms of the two drugs, thus not producing overlapping toxic reactions (21). Overall, compared to traditional neoadjuvant chemotherapy alone throughout the perioperative period, neoadjuvant chemoimmunotherapy is safer and limits the augmentation of adverse drug reactions. Therefore, neoadjuvant chemoimmunotherapy may be a safe treatment modality.

In conclusion, neoadjuvant chemoimmunotherapy not only has better efficacy than chemotherapy in stage III NSCLC but also has a reliable safety profile, which is an option for preoperative neoadjuvant treatment of stage III NSCLC. However, this was a small-sample retrospective clinical study with limited results; therefore, more large-sample, multicenter, prospective clinical studies are needed to verify the conclusions.

## Data availability statement

The original contributions presented in the study are included in the article/supplementary material. Further inquiries can be directed to the corresponding authors.

## Ethics statement

The studies involving humans were approved by Ethics Committee of Zhongshan City People’s Hospital (Zhongshan, China). The studies were conducted in accordance with the local legislation and institutional requirements. The participants provided their written informed consent to participate in this study.

## Author contributions

SHZ: Writing – original draft, Data curation, Formal Analysis, Investigation. YLiu: Data curation, Formal Analysis, Writing – original draft. KJL: Data curation, Investigation, Methodology, Writing – original draft. JKZ: Conceptualization, Supervision, Validation, Writing – review & editing. HLL: Investigation, Project administration, Supervision, Validation, Writing – review & editing. YMW: Project administration, Supervision, Validation, Writing – review & editing. HYY: Investigation, Supervision, Validation, Writing – review & editing. YLiang: Funding acquisition, Project administration, Supervision, Validation, Writing – review & editing. JJZ: Conceptualization, Data curation, Project administration, Validation, Writing – review & editing. WZH: Conceptualization, Funding acquisition, Methodology, Project administration, Writing – original draft, Writing – review & editing.
